# Do you pay to go private?: a single centre comparison of induction of labour and caesarean section rates in private versus public patients

**DOI:** 10.1186/s12884-020-03443-4

**Published:** 2020-12-01

**Authors:** Simon Craven, Fionnuala Byrne, Rhona Mahony, Jennifer M. Walsh

**Affiliations:** National Maternity Hospital, Holles Street, Dublin 2, Ireland

**Keywords:** Caesarean section, Induction of labour, Public versus private healthcare

## Abstract

**Background:**

The aim of this study was to compare rates of induction and subsequent caesarean delivery among nulliparous women with private versus publicly funded health care at a single institution. This is a retrospective cohort study using the electronic booking and delivery records of nulliparous women with singleton pregnancies who delivered between 2010 and 2015 in an Irish Tertiary Maternity Hospital (approx. 9000 deliveries per annum).

**Methods:**

Data were extracted from the National Maternity Hospital (NMH), Dublin, Patient Administration System (PAS) on all nulliparous women who delivered a liveborn infant at ≥37 weeks gestation during the 6-year period. At NMH, all women in spontaneous labour are managed according to a standardised intrapartum protocol. Twenty-two thousand two hundred thirty-two women met the inclusion criteria. Of these, 2520 (12.8%) were private patients; the remainder (19,712; 87.2%) were public. Mode of and gestational age at delivery, rates of and indications for induction of labour, rates of pre-labour caesarean section, and maternal and neonatal outcomes were examined. Rates of labour intervention and subsequent maternal and neonatal outcomes were compared between those with and without private health cover.

**Results:**

Women attending privately were more than twice as likely to have a pre-labour caesarean section (12.7% vs. 6.5%, RR = 2.0, [CI 1.8–2.2])); this finding persisted following adjustment for differences in maternal age and body mass index (BMI) (adjusted relative risk 1.74, [CI 1.5–2.0]). Women with private cover were also more likely to have induction of labour and significantly less likely to labour spontaneously. Women who attended privately were significantly more likely to have an operative vaginal delivery, whether labour commenced spontaneously or was induced.

**Conclusions:**

These findings demonstrate significant differences in rates of obstetric intervention between those with private and public health cover. This division is unlikely to be explained by differences in clinical risk factors as no significant difference in outcomes following spontaneous onset of labour were noted. Further research is required to determine the roots of the disparity between private and public decision-making. This should focus on the relative contributions of both mothers and maternity care professionals in clinical decision making, and the potential implications of these choices.

## Background

Ireland has the highest birth rate among European Union countries, with 63,900 live births recorded during 2016, a rate of 13.5 births for every 1000 of the population [1]. Currently, maternity services in Ireland are predominantly hospital based, with over 99% of births occurring within a hospital setting; pregnancy is the largest single reason for admission to hospital [2]. The current Irish model of maternity care was originally devised under the 1954 Mother and Infant Care Scheme. This granted women resident in Ireland access to free antenatal care, continued up to 6 weeks postpartum. This model of care is a combined one, with women being cared for by both a general practitioner (GP) in the community and a maternity health care provider in a hospital clinic as pregnancy progresses. These public obstetric patients may see a range of doctors or midwives at clinics, and will only have a doctor at the birth if required. While the majority of Irish women avail of this model of care, a significant proportion choose private care (within a public hospital setting) - whereby antenatal visits are with a chosen consultant obstetrician, who will personally attend at delivery unless unavailable.

It is well reported that rates of caesarean section (CS) are increasing [3]. A number of factors contribute to rising CS rates. These include: increasing maternal age, obesity, medical co-morbidities, elective repeat CS and CS at maternal request [4]. In addition, there is increasing evidence for an association between private funding and CS rates. Variation in intervention rates by socioeconomic indicators, such as type of health care coverage, suggests that obstetric practice may not be solely driven by case criteria [5, 6].

There is contention, whether private care within publicly funded hospitals incurs a higher rate of interventions, and whether these costly interventions, in terms of investigations, operations and perioperative care, are justified by the risk profile of the patients [7–9].

At our institution, the management of women in labour is standardised and all spontaneous nulliparous labours are actively managed according to an intrapartum protocol [10] (see detailed intrapartum management plan in Methods section). This management should, in theory, minimise any potential healthcare provider bias for those women having their first baby who labour spontaneously, but does not affect the decision to induce labour or perform a caesarean section prior to the onset of labour.

The aim of this study was to compare rates of obstetric intervention among women with private versus public health care in an integrated maternity hospital setting. While allowing for adjustments in maternal age and BMI, differences in rates of caesarean delivery, induction of labour and spontaneous onset of labour were examined.

We hypothesised that obstetric interventions would be higher among women with private healthcare coverage.

## Methods

### Study design

This is a retrospective cohort study using the electronic booking and delivery records of nulliparous women with singleton pregnancies who delivered between January 2010 and December 2015 at the National Maternity Hospital, Dublin, Ireland. This is a tertiary level referral institution in which approximately 9000 women deliver annually. We excluded preterm deliveries (less than 37 weeks gestation), multiple pregnancies and stillbirths. We also excluded those who did not have private or public data recorded (*n* = 679). Study participants were subdivided into two groups based on whether they booked for private or public obstetric care. Private patients choose their own obstetrician and either self-refer or are referred by their general practitioner. The named obstetrician provides continuity of care throughout the antenatal, intrapartum and postnatal period with occasional cross-cover by a nominated obstetrician colleague. Public patients are booked under a consultant obstetrician team and care is shared between midwives, the general practitioner and the obstetric team including doctors in training.

### Intrapartum management

Nulliparous women attending in spontaneous labour at term with a singleton cephalic presentation are managed according to a standardised intrapartum active management protocol [10]. This protocol has been reported in previously published work [11], and comprises early diagnosis of labour followed by early artificial rupture of membranes (AROM). Once labour is diagnosed, two-hourly assessment of cervical dilatation is carried out. If dilatation does not progress at a rate of 1 cm/h oxytocin augmentation is instituted. In the absence of fetal distress, and if delivery is not imminent within 12 h of admission to the labour ward, a caesarean section is performed. When induction of labour is indicated, either dinoprostone gel is administered or an AROM is carried out, depending on modified Bishop score [12]. Mechanical methods of induction are not utilised. If labour has not begun within 20–24 h, an intravenous oxytocin induction is initiated. Induced labour is managed according to active management principles. Oxytocin for both induction and augmentation is administered in a concentration of 10 IU/L, commencing at a rate of 5 mU/ min and increasing by 5 mU/min every 15 min to a maximum dosage of 30 mU/min, unless uterine contraction frequency exceeds seven in 15 min. All labour details are documented on standardised partograms by the supervising midwife and recorded prospectively on the National Maternity Hospital (NMH) Patient Administration System (PAS) computerised database; these data on labour outcomes are subject to continuous audit of anonymised data.

### Data collection

We sought to compare rates of obstetric intervention in those women attending for private versus public obstetric care. Relevant descriptive statistics were obtained for the study population. Mode of and gestational age at delivery, rates of and indications for induction of labour, rates of pre-labour caesarean section, and maternal and neonatal outcomes were examined.

### Statistical analysis

Data were assessed for normality using Shapiro Wilk and P-P plot. Non-parametric data were log transformed and normality checked prior to analysis. Comparison of means within groups of patients was accomplished with the independent samples t test. Comparisons between non-continuous data were made by using a chi-square test. Multiple logistic regression analysis was used to produce a multivariate regression model to adjust for the potential contribution of demographic confounders. Statistical significance was set at *P* < 0.05. Statistical analysis was performed using SPSS software (version 24, SPSS Chicago, IL, USA).

## Results

During the 2010–2015 study period, 22,232 nulliparous women met the inclusion criteria. Of these, 2520 (11.3%) were private patients; the remainder 19,712 (88.7%) were public.

Characteristics of the study population are outlined in Table 1.

**Table 1:** Characteristics of the Study Population

	Private ***N*** = 2520	Public ***N*** = 19,712
**Maternal age (years)**	35.89 ± 3.8	32.02 ± 5.1
**BMI (kg/m**^**2**^**)**	23.48 ± 3.8	24.81 ± 4.4
**GA (weeks)**	39.78 ± 1.2	39.85 ± 1.3
**Birthweight (grams)**	3577.6 ± 460	3509 ± 471

Characteristics of the Study Population at first antenatal consultation. Results are shown as mean+/− SD

*BMI* Body mass index, *GA* Gestational age

Overall, women attending for private care were older with lower body mass index (BMI) than public patients, and delivered at an earlier gestational age (Table 1).

In total, 5214 women were delivered by CS, an overall rate of 22.8%.

Women attending privately were more than twice as likely to have a pre-labour CS (12.7% vs. 6.5%, RR = 2.0, [CI 1.8–2.2]); this finding persisted following adjustment for differences in maternal age and BMI (adjusted relative risk 1.74, [CI 1.5–2.0]). Women with private cover were also more likely to have an induction of labour and significantly less likely to labour spontaneously. Women with private cover who were induced were more likely to be delivered by CS (35% vs. 30.6%); however when differences in maternal BMI and age were accounted for in the multivariate regression model, no significant difference between private and public patients in CS rates following induction of labour remained. Women who attended privately were also significantly more likely to have an operative vaginal delivery, whether labour commenced spontaneously or was induced (Table 2).

**Table 2:** A comparison of onset of labour, mode of delivery and maternal and neonatal outcomes between private and public patients

	Private (***n*** = 2520)	Public (***n*** = 19,712)	Relative Risk 95% CI	Adjusted Relative Risk 95% CI
*Onset*				
**Pre-labour CS**	319	1210	1.97	1.74
			1.76–2.18	1.48–2.04
**IOL**	988	6565	1.42	1.30
			1.31–1.53	1.17–1.44
**SOL**	1213	11,937	0.64	0.64
			0.56–0.66	0.58–0.71
*Mode of delivery*				
**CS (Total)**	773	4234	1.06	1.37
			1.05–1.08	1.27–1.53
**CS following SOL**	112	1101	1.01	0.97
			0.99–1.03	0.76–1.23
**CS following IOL**	342	2018	1.02	1.1
			1.01–1.04	0.89–1.26
**OVD**	710	4365	1.64	1.36
			1.5–1.8	1.20–1.53
**OVD following SOL**	411	2850	1.6	1.3
			1.42–1.80	1.11–1.51
**OVD following IOL**	299	1515	1.61	1.37
			1.39–1.85	1.13–1.68
*Outcomes*				
**OASIS**	27	482	0.46	0.38
			0.32–0.66	0.25–0.58
**PPH >500mls**	441	3428	1.01	0.93
			0,91–1.11	0.81–1.05
**Cord pH < 7.10**	48	387	0.98	0.98
			0.82–1.19	0.72–1.34
**Neonatal unit admission**	143	1226	0.92	0.92
			0.72–1.02	0.77–1.10

*CS* Caesarean section, *IOL* Induction of labour, *SOL* Spontaneous onset of labour, *OVD* Operative vaginal delivery, *OASIS* Obstetric anal sphincter injury, *PPH* Post-partum haemorrhage

Women attending privately were less likely to have an obstetric anal sphincter injury (OASIS) following vaginal delivery; no differences in the incidence of post-partum haemorrhage (PPH) were noted. A similar proportion of private and public patients had cord pH levels checked at birth (1025/2520, 40.7% vs. 8041/19,712, 40.8%, *p* = 0.9); no difference in cord pH levels were observed. Similarly, no difference in admission rates to the neonatal unit were seen (Table 2).

## Discussion

This study contributes to a growing body of evidence supporting an association of increased obstetric intervention in women with private health care cover. This is well reported, particularly in Australia [7, 13–15]. A 2013 study by Murphy and Fahey identifies similar findings in a separate tertiary hospital in Ireland [16]. Our data demonstrates that women in their first pregnancy attending an obstetrician privately in the National Maternity Hospital, Dublin, were more than twice as likely to have a pre-labour caesarean section when compared to public patients, and 1.3 times more likely to have their labour induced. Private patients were also significantly more likely to have an operative vaginal delivery, both in spontaneous and induced labours. These findings could not be explained by differences in the main baseline predictors of obstetric outcome, namely maternal BMI and age. A difference in the incidence of significant perineal damage following childbirth was observed, but no difference in primary haemorrhage rates or neonatal outcome between the two groups was seen.

The disparity in caesarean delivery rates between women with public versus private health care cover has previously been well described nationally and internationally [5, 6, 17, 18]. Turner et al. in 2016 assessed factors influencing national variation in caesarean delivery rates, and concluded that private care was an important predictor of delivery by caesarean. They noted that the proportion of variation was higher for elective CS than emergency CS, suggesting that variation was more likely influenced by antenatal decision making [6]. Similar findings have been demonstrated in an Australian population, with primigravidae attending for private obstetric care significantly more likely to deliver by assisted vaginal or caesarean delivery [18].

These findings are clinically important. Labour interventions, such as a lower threshold to induce labour, or to perform a primary CS are costly, both economically and medically. While the cost to the health service is generally higher in caesarean when compared to spontaneous vaginal deliveries, it is the impact on the woman’s health, and future reproductive career that is most profound [19, 20]. The dramatic rise in the incidence of abnormally invasive placenta is directly linked to rising CS rates [21, 22]; indeed it has even been suggested that women with a primary elective caesarean section without labour are more likely, compared with those undergoing primary emergency caesarean section with labour, to develop an accreta in a subsequent pregnancy [23].

Importantly, in our study, no difference between private and public patients in CS rates following onset of labour was noted. This finding is consistent with that reported in the article by Murphy and Fahey [16]. Our results may be accounted for by the standardised management of labour at the National Maternity Hospital, irrespective of healthcare coverage. This suggests that there may be an element of clinician bias toward private patients which results in a lower threshold to induce labour, or perform a caesarean section prior to labour, but that this bias may be removed once a standardised protocol for labour management is in place in an institution. In order test this hypothesis, a study exploring CS rates before and after implementation of a standardised protocol should be undertaken.

### Strengths and limitations

Our results are limited by the lack of data on pre-existing medical problems, socioeconomic factors and antenatal complications which may have influenced decision making in the antenatal period. Previous studies have shown that private patients, despite being older, tend to be healthier with fewer reported pre-existing medical conditions [16]. In this study, when the confounding factors of maternal age and BMI were corrected for, although the difference was attenuated, the difference between groups persisted. Additional work is required, not only to further elucidate the influence of such potential confounders, but also to determine if such influence is warranted or justified by maternal or neonatal outcomes.

There is ongoing debate whether rates of caesarean section could or should be lowered, and whether the associated complications are justified [24]. Most health professionals agree that the management of a woman’s first birth is likely to have the biggest impact on overall rates of caesarean section. There is little data in the literature to date to suggest that a standardised management protocol for first time mothers in spontaneous labour appears to eliminate any influence that private healthcare coverage may have on delivery outcomes in this cohort. This is important, as ultimately safe prevention of the primary caesarean is of paramount importance to control the internationally rising caesarean delivery rate. Major obstetric haemorrhage related to abnormal placentation or placenta accreta spectrum is rapidly becoming the most significant cause of maternal morbidity and mortality worldwide, and will be an increasing challenge in years and decades to come as the number of women with a uterine scar increases. Variation in intervention rates by socioeconomic indicators, such as type of health care coverage, suggests that obstetric practice may not be solely driven by case criteria. Although speculative, it is likely that private patients are provided with greater choice in relation to a scheduled caesarean section [16, 25]. It is debatable whether this is actually in the woman’s best interest, particularly when it comes to the next birth [26–29]. In the U. S, the American College of Obstetricians and Gynecologists (ACOG) state that the “Committee on Obstetric Practice believes that in the absence of maternal or fetal indications for caesarean delivery, a plan for vaginal delivery is safe and appropriate and should be recommended to patients” [30]. The World Health Organisation (WHO) statement on caesarean section rates states that “there is no evidence showing the benefits of caesarean delivery for women or infants who did not require the procedure” [31]. The incidence of caesarean delivery on maternal request and its contribution to the overall increase in the caesarean delivery rate has not been quantified clearly. In the United States the caesarean section rate for women with no medical indication has been rising since 1991 and is currently at 11.2% of the total caesarean section rate performed in nulliparous women [32]. In 2001, in the UK 21% of all births were by caesarean section and, of these, 5% of women requested caesarean section for no medical reason and 1.4% had caesarean section on maternal request [33].

Clear and robust audit and reporting of rates of, and indications for caesarean delivery are of paramount importance in order to fully determine both the factors influencing the decision making process, and also the implications for maternal, neonatal and public health. Using mixed methods research could provide a valuable insight regarding the decision to perform a caesarean delivery in both public and private patients. In 2015, WHO proposed the use of the Robson classification (also known as the 10-group classification) as a global standard for assessing, monitoring and comparing caesarean section rates both within healthcare facilities and between them. This classification system inherently captures many important risk factors for obstetric intervention [31]. Using this framework, qualitative research can be undertaken to investigate the different dynamics in different subgroups, with varying levels of risk for caesarean delivery. Such research would ideally focus on clinical decision-making and the influence of the personal preferences of women versus those of maternity health care professionals.

## Conclusion

In conclusion, these data suggest that there are significant differences in the management of both private and public patients. Although these trends are undoubtedly impacted by differences in obstetric profiles, this work implies that health care coverage status may be an independent risk factor for intervention. Further audit and research is needed to determine the roots of the disparity between private and public decision-making, and the potential clinical implications for both mothers and their infants.

## Data Availability

The datasets used and/or analysed during the current study are available from the corresponding author on reasonable request.

## Abbreviations

CS: cesarean section
GP: general practitioner
AROM: artifical rupture of membranes
BMI: body mass index
GA: gestational age
OASIS: obstetric anal sphincter injury
ACOG: American College of Obstetricians and Gynecologists
IOL: induction of labour
SOL: spontaneous onset of labour
OVD: operative vaginal delivery
PPH: post-partum haemorrhage
